# Use of an Absorbable Synthetic Polymer Dural Substitute for Repair of Dural Defects: A Technical Note

**DOI:** 10.7759/cureus.2127

**Published:** 2018-01-29

**Authors:** Philip Schmalz, Christoph Griessenauer, Christopher S Ogilvy, Ajith J. Thomas

**Affiliations:** 1 Department of Neurosurgery, University of Alabama at Birmingham; 2 Department of Neurosurgery, Geisinger Medical Center; 3 Neurosurgery, BIDMC Harvard Medical School

**Keywords:** dura mater, polydioxanone, polyglycolic acid, cerebrospinal fluid leak, wound healing

## Abstract

Repair of the dura after cranial neurosurgery can present a technical challenge and, for certain neurosurgical procedures, is critical to prevent cerebrospinal fluid leak and associated wound complications. Multiple options exist for dural repair, including the patient’s own tissues, bovine collagen-derived commercially available grafts, as well as newer, entirely synthetic graft materials. This is the first report of surgical experience with a new synthetic and absorbable dura substitute which has recently gained Food and Drug Administration (FDA) approval.

Four patients underwent dural reconstruction with a new graft material after cranial neurosurgery when the dura was unrepairable directly.

The synthetic graft material demonstrated satisfactory surgical qualities, was effective in dural repair, and no complications were attributable to the graft.

Dural repair after craniotomy is an often desirable surgical outcome in neurosurgery. Surgeons seeking new graft materials may find this new absorbable, entirely synthetic material favorable for dural repair.

## Introduction

Repair of the dura mater after cranial and spinal neurosurgery can often present a technical challenge. At the termination of many routine supratentorial operations, and even more commonly during posterior fossa and skull base procedures, the surgeon is confronted with unrepairable dura and a high risk of cerebrospinal fluid (CSF) leak. Dural defects are caused by shrinkage through bipolar coagulation, dural attenuation, or laceration during exposure. Meningioma surgery in particular often requires excision or destruction of the dura to obtain a satisfactory tumor resection and achieve the desired surgical result. Commonly, tension-free repair is unfeasible in these circumstances and patch grafting of the dura is required for watertight closure. Multiple options exist for the repair of a dural defect including autografts such as pericranium or fascia, allo- or xenografts derived from human or bovine fascia, pericardium, or skin, and commercially processed graft material often prepared from bovine collagen sources or human cadaveric tissues. More recently, entirely synthetic and absorbable dura substitutes have received Food and Drug Administration (FDA) approval. These include Cerafix Dura Substitute (Acera Surgical, St. Louis, MO, USA) and Ethisorb (Codman, Raynham, MA, USA) [1,2]. These products have the advantage of ready availability, can be cut to shape, and as they are manufactured can be produced with uniform handling characteristics. Furthermore, as they are not derived from biological sources, there is no risk of disease transmission. We present the first single-center experience of four patients who underwent craniotomy with dural repair using Cerafix Dura Substitute, a newly FDA-approved, commercially available porous polymer matrix designed for dural repair. This research was unfunded and was conducted independently of any industry guidance or support.

## Technical report

Cerafix Dura Substitute is a synthetic, porous polymer matrix composed of spun poly(lactic-co-glycolic acid) (PGLA) and poly-p-dioxanone (PDS) that has been FDA-approved for the treatment of dural defects of 12.5 cm^2^ or less in area [1]. It is available in sizes ranging from 2.5 x 2.5 cm, up to 10 x 12.5 cm, and may be cut to shape. Prior to implantation, it was hydrated according to package instructions. The graft was then measured and cut to the appropriate size and shape of the dural defect, plus an approximately 1 cm overlap as specified in the manufacturers' instructions. Placed over the defect, it was sutured in place using running 4-0 Neurolon suture (Ethicon US, Somerville, NJ, USA). This study was conducted in accordance with institutional guidelines and with the approval of the Institutional Review Board.

Four patients were treated via elective supratentorial craniotomy at a single institution over a six-week period in November and December of 2016. In all patients at the time of closure, the dura was deemed unable to be closed by primary repair in a watertight fashion. Cerafix was used to close the dural defect. It was hydrated, measured and cut to the appropriate size and shape of the dural defect, plus an approximately 1 cm overlap. It was sutured in place using running 4-0 Neurolon suture. Care was taken both to create a tension-free repair and to establish a watertight seal.

**Figure 1:**
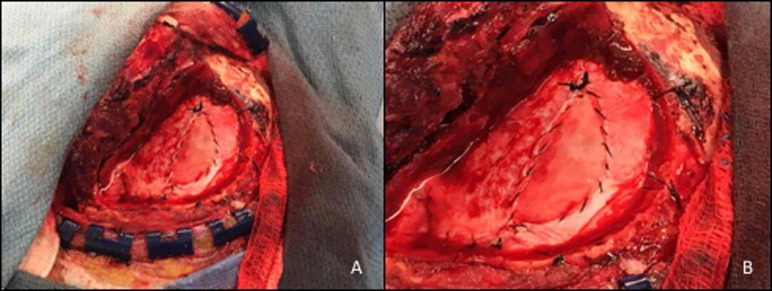
Dural defect repaired with synthetic dural substitute. Case 4: A 59-year-old man underwent a frontotemporal craniotomy for left medial sphenoid wing meningioma. The dural defect was closed using Cerafix secured with running 4-0 Neurolon suture (Panels A and B, enlarged).

The first patient (Case 1), a 63-year-old female, presented with an 8 mm unruptured left middle cerebral artery aneurysm and underwent a left pterional craniotomy and clipping of this aneurysm. Two patients (Cases 2 and 3), both female, aged 56 and 67, who presented with glioblastoma underwent craniotomy and resection of tumor. Both patients were treated postoperatively with routine concurrent chemo- and radiotherapy according to the regimen described by Stupp, et al. [3]. Lastly, a 59-year-old man (Case 4) presented with a left medial sphenoid wing meningioma, WHO Grade II, and was treated with a left frontotemporal craniotomy and tumor resection (Figure 1). This patient did not receive postoperative radiotherapy. In all of the above cases, the dura was either lacerated, required resection to obtain complete tumor removal, or had retracted such that a tension-free direct primary repair was deemed unlikely to succeed. Available Cerafix sizes were sufficient to close all defects without the need to use multiple grafts. Repair of the dura in a watertight and tension-free manner with Cerafix was successful in all cases. The material was soft, pliable, and was able to withstand surgical manipulation and hold sutures. In all patients wound healing proceeded without complication. There was no clinical evidence of CSF leak or the development of a pseudomeningocele. No patient reported symptoms suggestive of chemical meningitis such as fever, neck stiffness, or nausea and vomiting. One patient with glioblastoma reported a transient headache five weeks after operation which self-resolved. A second patient with glioblastoma developed a spontaneous retinal detachment during radiotherapy which required surgical repair. Further radiotherapy was suspended in this patient. No patient developed an infection. Routine postoperative computed tomography (CT) and contrasted magnetic resonance imaging (MRI) were performed in those patients diagnosed with glioblastoma. In these patients, there was no imaging evidence of persistent fluid collection to suggest CSF leakage or pseudomeningocele formation, nor was there evidence of meningeal enhancement to suggest the development of subclinical chemical meningitis. All patients were followed clinically for a minimum of six months and no postoperative complications attributable to the graft were observed during this time.

## Discussion

Here, we present the first case series of patients that had postoperative dural defects closed with Cerafix Dura Substitute, a novel, synthetic, porous polymer matrix composed of spun PGLA and PDS that has been recently FDA-approved. The material had satisfying handling capabilities and was used without complications in all cases.

While dural reconstruction is often not widely perceived as a critical step in complex cranial neurosurgery, closure of the dura mater is often a desirable and at times a key step towards a successful surgical outcome. Postoperative wound complications can carry significant morbidity, and experienced neurosurgeons are wise to pay attention to dural reconstruction. Dural closure is particularly critical after procedures in the posterior fossa or skull base where CSF leak risk is high. CSF leakage can result in complications with significant morbidity, including infection and the requirement for CSF diversion by lumbar or external ventricular drain. When less invasive methods fail, operative repair of these leaks can cause further surgical morbidity, pain, and patient dissatisfaction.

Multiple options exist for the repair of a dural defects including autografts, allo- or xenografts, and commercially processed graft material prepared from bovine collagen sources or human cadaveric tissues [4]. More recently, nonbiological materials have been developed as dura substitute, including absorbable polymers such as those commonly used in sutures, as well as nonabsorbable inert polymers such as aliphatic polyurethane [5,6]. Autograft materials have a number of advantages, including biological compatibility, availability, and cost. However, their procurement often requires additional or enlarged incisions with the attendant surgical risk, as well as postoperative pain and discomfort. Autograft material can vary greatly in quality due to host tissue properties or harvesting technique. Autograft harvest may be inconvenient, particularly if the need for a graft was not anticipated at the start of surgery and a donor site is not readily accessible. Allografts and xenografts have the advantage of surgical convenience and ready availability, but can be quite costly. These materials are native tissues and, like autograft, can vary substantially in quality and handling characteristics. Though uncommon, it should also be noted that allo- and xenografts, carry a risk of transmission of infectious disease. Several cases of transmission of Creutzfeldt–Jakob disease have been reported with the use of cadaveric human dura [7,8]. Bovine pericardium has previously been used for the prevention of adhesions after decompressive craniectomy to facilitate cranioplasty [9]. A commercially available synthetic material may also serve well in this role. Commercially available processed dural substitutes have been produced for some time and are familiar to many neurosurgeons. These products are derived most often from bovine or other animal collagen sources. Some of the more familiar include Durepair (Medtronic, Minneapolis, MN, USA), DuraGen (Integra North America, Plainsboro, NJ, USA), Dura-Guard (Baxter, Deerfield, IL, USA), among others [10,11]. They have the advantage of uniformity and availability. These products come in a variety of sizes and can be cut to shape. Not all are designed to hold suture, however. Also, as they are derived from living tissues, these products require post-procurement processing and, like all non-native tissues, pose a risk of adverse reaction to foreign biological material [12-14]. Like Cerafix, these materials are designed to be gradually absorbed by tissue ingrowth and replacement of the graft material with the patient's native tissues. Non-human studies and clinical observation of these biological materials have demonstrated tissue ingrowth, however this has not yet been observed in Cerafix with human subjects. Cerafix is a novel, synthetic, porous polymer matrix formed from PGLA/PDS fibers in nonwoven sheets. These materials may be more familiar to surgeons in the form of Vicryl and PDS sutures (Ethicon US, Somerville, NJ, USA), respectively. The first, PGLA, is designed to provide a matrix for tissue ingrowth and graft replacement much the same as with absorbable suture. The second, PDS, is designed to provide a watertight barrier during the absorptive process. The material is soft, nonfriable, compliant, and absorbable. Cerafix has been shown to be comparable to other commercially available dural substitutes [1]. Unlike some other commercially available dural substitutes, Cerafix is designed to function as a suturable dural patch. In the small series presented, the material performed as expected and no complications were attributable to the graft during a minimum follow-up duration of six months.

## Conclusions

Here we report a single-surgeon experience in four patients with a newly-approved synthetic dural substitute, Cerafix. This product received FDA approval in March, 2016 and neurosurgical experience with it is limited. In the above four cases, the material performed satisfactorily and there was no evidence of a complication attributable to the graft. Closure of dural defects with patch grafts is a common need in cranial neurosurgery, and this report may be of interest to those seeking alternative dural substitute material.
